# Genomics Meets Glycomics—The First GWAS Study of Human N-Glycome Identifies HNF1α as a Master Regulator of Plasma Protein Fucosylation

**DOI:** 10.1371/journal.pgen.1001256

**Published:** 2010-12-23

**Authors:** Gordan Lauc, Abdelkader Essafi, Jennifer E. Huffman, Caroline Hayward, Ana Knežević, Jayesh J. Kattla, Ozren Polašek, Olga Gornik, Veronique Vitart, Jodie L. Abrahams, Maja Pučić, Mislav Novokmet, Irma Redžić, Susan Campbell, Sarah H. Wild, Fran Borovečki, Wei Wang, Ivana Kolčić, Lina Zgaga, Ulf Gyllensten, James F. Wilson, Alan F. Wright, Nicholas D. Hastie, Harry Campbell, Pauline M. Rudd, Igor Rudan

**Affiliations:** 1 Glycobiology Laboratory, Genos Ltd., Zagreb, Croatia; 2 Department of Biochemistry and Molecular Biology, University of Zagreb, Faculty of Pharmacy and Biochemistry, Zagreb, Croatia; 3 Medical Research Council Human Genetics Unit, Institute of Genetics and Molecular Medicine, Western General Hospital, Edinburgh, United Kingdom; 4 National Institute for Bioprocessing Research and Training, Dublin-Oxford Glycobiology Lab, Conway Institute, University College Dublin, Dublin, Ireland; 5 Conway Institute, University College Dublin, Dublin, Ireland; 6 Gen Info Ltd., Zagreb, Croatia; 7 Medical School, University of Zagreb, Zagreb, Croatia; 8 Centre for Population Health Sciences, The University of Edinburgh Medical School, Edinburgh, United Kingdom; 9 School of Public Health and Family Medicine, Capital Medical University, Beijing, China; 10 Graduate School of the Chinese Academy of Sciences, Beijing, China; 11 Croatian Centre for Global Health, University of Split Medical School, Split, Croatia; 12 Department of Genetics and Pathology, Rudbeck Laboratory, Uppsala University, Uppsala, Sweden; Georgia Institute of Technology, United States of America

## Abstract

Over half of all proteins are glycosylated, and alterations in glycosylation have been observed in numerous physiological and pathological processes. Attached glycans significantly affect protein function; but, contrary to polypeptides, they are not directly encoded by genes, and the complex processes that regulate their assembly are poorly understood. A novel approach combining genome-wide association and high-throughput glycomics analysis of 2,705 individuals in three population cohorts showed that common variants in the Hepatocyte Nuclear Factor 1α (*HNF1α*) and fucosyltransferase genes *FUT6* and *FUT8* influence N-glycan levels in human plasma. We show that HNF1α and its downstream target HNF4α regulate the expression of key fucosyltransferase and fucose biosynthesis genes. Moreover, we show that HNF1α is both necessary and sufficient to drive the expression of these genes in hepatic cells. These results reveal a new role for HNF1α as a master transcriptional regulator of multiple stages in the fucosylation process. This mechanism has implications for the regulation of immunity, embryonic development, and protein folding, as well as for our understanding of the molecular mechanisms underlying cancer, coronary heart disease, and metabolic and inflammatory disorders.

## Author Summary

By combining recently developed high-throughput glycan analysis with genome-wide association study, we performed the first comprehensive analysis of common genetic polymorphisms that affect protein glycosylation. Over half of all proteins are glycosylated; but, due to difficulties in glycan analysis and the absence of a genetic template for their synthesis, knowledge about the complex processes that regulate glycan assembly is still limited. We demonstrated that HNF1α regulates the expression of key fucosyltransferase and fucose biosynthesis genes and acts as a master regulator of plasma protein fucosylation. Proper protein fucosylation is essential in numerous processes including inflammation, cancer, and coronary heart disease, thus the identification of a master regulator of plasma protein fucosylation has important implications for understanding both normal biological functions and disease processes.

## Introduction

Glycosylation is a post-translational modification that enriches protein complexity and function. Over half of all known proteins are modified by covalently bound glycans, which are important for normal physiological processes, including protein folding, degradation and secretion, cell signalling, immune function and transcription [1]–[4]. Configuration and composition of attached glycans significantly change the structure and activity of polypeptide portions of glycoproteins [5] and since this process is not template driven, complexity of the glycoproteome is estimated to be several orders of magnitude greater than for the proteome itself [6]. Disregulation of glycosylation is associated with a wide range of diseases, including cancer, diabetes, cardiovascular, congenital, immunological and infectious disorders [1], [3], [7]. Enzymes that are involved in glycosylation may therefore be promising targets for therapy [8]. The most prominent example of the importance of N-glycosylation is the group of rare diseases named congenital disorders of glycosylation [9] where different mutations in the biosynthesis pathway of N-glycans cause significant mortality and extensive motor, immunological, digestive and neurological symptoms [10], [11].

Due to experimental limitations in quantifying glycans in complex biological samples, our understanding of the genetic regulation of glycosylation is currently very limited [12]. However, recent technological advances have allowed reliable, high-throughput quantification of N-glycans [13], which now permits investigation of the genetic regulation and biological roles of glycan structures and brings glycomics into line with genomics, proteomics and metabolomics [14]. Recently we completed the first comprehensive population study of human plasma N-glycome which revealed variability that by far exceeds the variability of proteins and DNA [15]. However, within a single individual composition of plasma glycome is rather stable [16] and environmental factors have limited impact on the majority of glycans [17]. Specific altered glyco-phenotypes that can be associated with specific pathologies were also identified to exist in a population [18].

Variations in glycosylation are of great physiological significance as alterations in glycans significantly change the structure and function of polypeptide parts of glycoproteins [5]. A particularly interesting element of protein glycosylation is the addition of fucose to non-reducing ends of N-glycans. Fucose is a relatively novel sugar in evolutionary terms with two important structural features that distinguishes it from all other mammalian six-carbon monosaccharides; it lacks a hydroxyl group on the carbon at the 6-position and is the only monosaccharide that is in the L-configuration. The conversion of GDP-mannose to GDP-fucose is catalyzed by two enzymes (GMD and FX) that display remarkable evolutionary conservation [19], [20]. On the other hand, the large family of genes that add fucose to proteins and lipids (fucosyltranferases, FUTs) has a very complex evolutionary history, including several more recent events specific to primates [21]. In mammals, fucose-containing glycans have important roles in blood transfusion reactions, in the selectin-mediated leukocyte-endothelial adhesion that initiates an inflammatory response, in host-microbe interactions, and numerous ontogenic events [10], [19]. Acute phase proteins have altered fucosylation in many diseases [22] and changes in the levels of fucosylated glycans have been shown to be associated with several important pathological processes, including cancer [23].

Hepatocyte nuclear factor 1α (HNF1α) and its downstream target HNF4α are transcription factors that regulate gene expression in both the liver and pancreas in a tissue-specific manner and are key regulators of metabolic genes [24]. Mutations in the encoding genes *HNF1α* and *HNF4α* cause Maturity Onset Diabetes of the Young (MODY) types 3 and 1 respectively [25], [26]. Recently, *HNF1α* single nucleotide polymorphisms (SNPs) have been associated with plasma C-reactive protein (CRP) [27], LDL cholesterol and gamma glutamyltransferase (GGT) [28], and coronary heart disease [29]. *HNF4α* variants have been associated with ulcerative colitis [30] and with the plasma concentrations of CRP and apolipoprotein A1 (APOA1) [31]. Currently there is little evidence to link these transcription factors with fucose metabolism and the upstream mechanisms regulating fucosylation pathways are unknown.

## Results

### Common variants in fucosyltransferase genes affect the relative proportions of plasma N-glycans

We performed the first systematic analysis of the genetic regulation of individual N-glycans in plasma from 2,705 individuals in three population cohorts, from Croatia and Scotland, which have previously been characterized in great detail [32]. Desialylated 2AB-labelled human plasma N-glycans were separated into 13 structurally related groups of glycans, referred to as DG1–DG13 (see Table S1 for a list of specific glycans found within each DG group) [13]. The concentration of plasma N-glycans measured in each of these groups was then expressed as a proportion of the total plasma N-glycome to obtain 13 quantitative variables in each examinee. All N-glycans contain two core N-acetylglucosamine (GlcNAc) residues, to which a “core” fucose can be α1,6-linked to the inner GlcNAc, which is directly linked to an asparagine residue on the protein. Additional fucose residues can be transferred to different positions on antennas that have been added to the core glycan structure (Table S1). Two further traits were derived from the original variables to calculate the percentage of glycan structures containing core (FUC-C) or antennary (FUC-A) fucose, yielding a total of 15 glycan traits for analysis.

We conducted a meta-analysis of genome-wide association study (GWAS) data for the fifteen plasma N-glycan traits measured in three population-based cohorts, CROATIA-VIS (n = 924), CROATIA-KORCULA (n = 898) and ORCADES (n = 737). Additive SNP effects were tested in each cohort independently and then combined in an inverse-variance weighted meta-analysis. The genome-wide significance threshold for the meta-analysis was set at 5×10^−08^.

**Figure 1:**
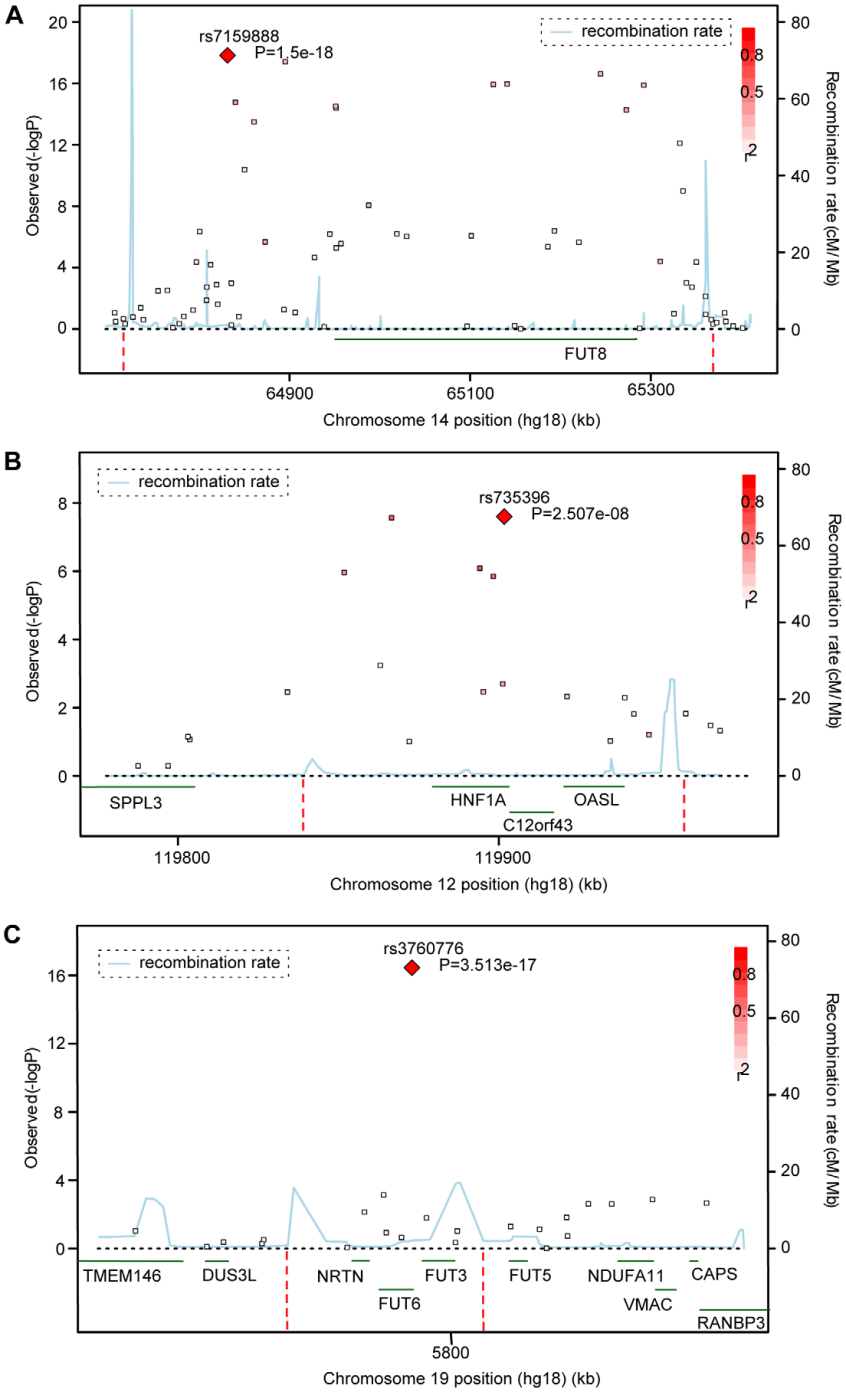
Significance plots. Significance plots for regions of interest from the meta-analysis of (a) DG1, (b) DG7 and (c) DG9. A region around the most significant genotyped SNP (represented by a red diamond) is displayed with the –log_10_ of the association p-values plotted against chromosome position. The degree of linkage disequilibrium between the most significant SNP and any SNP tested is indicated by a gradient of red shading. Recombination rate is displayed by a blue line with scale on the right-hand axis. Characterized genes in the region are represented with an arrow (showing the direction of transcription). The accompanying association interval as defined in the methods is marked by vertical red dotted lines.

**Figure 2:**
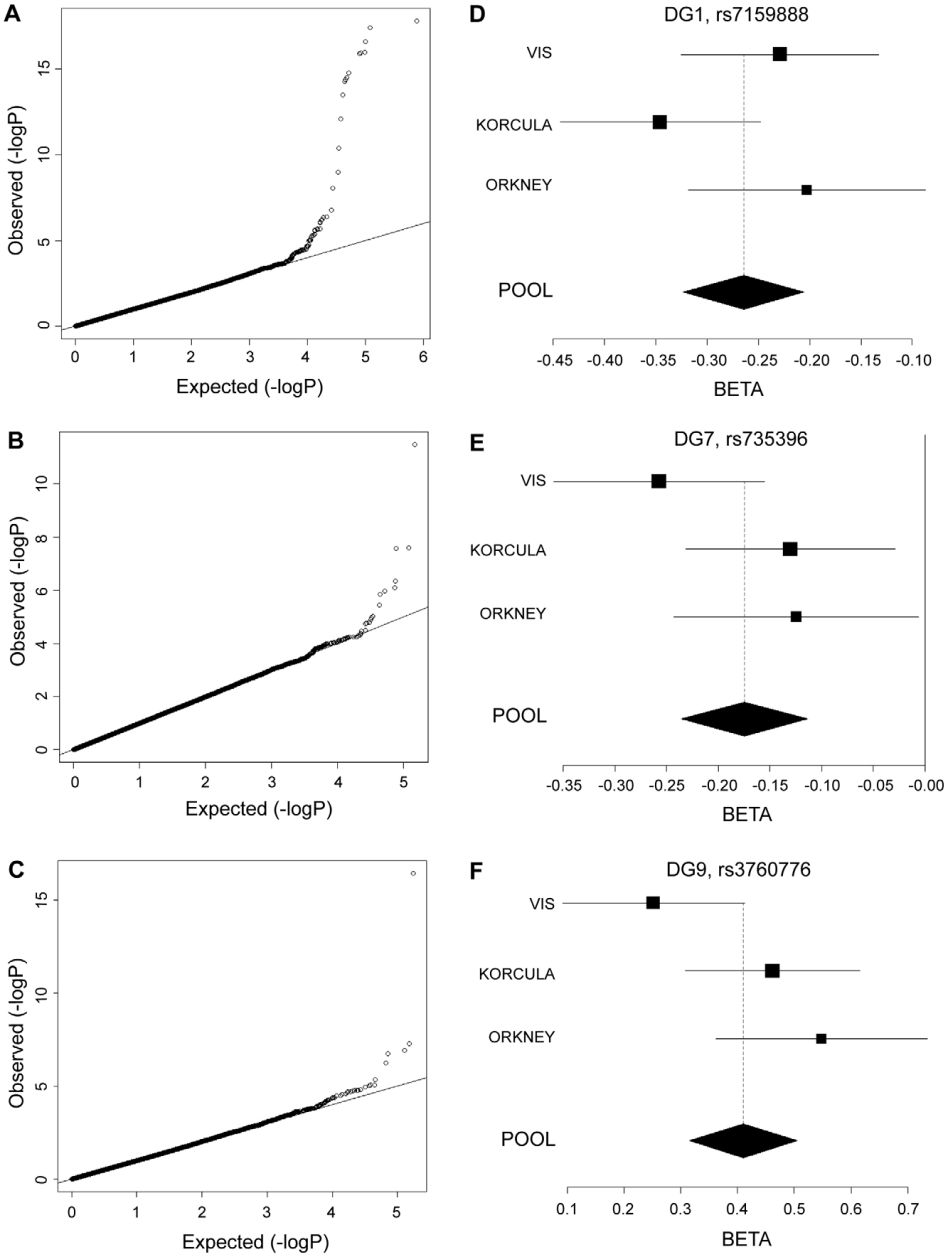
Quantile–quantile plots and forest plots. Quantile-quantile plots for test statistics (a–c) and forest plots of the most significant SNPs (d–f) from the meta-analysis of DG1, DG7, and DG9. −Log_10_ of the association p-values are plotted against −log_10_ of the expected p-value under the null hypothesis of no association for the meta-analysis. The effect size estimates for each individual population and the meta-analysis are shown along with their standard errors. The effect size presented is the β-coefficient, which represents a change in glycan levels measured in standard deviation units (adjusted for covariates) per copy of the allele modelled. The mean effect size estimates are represented by a square for individual cohorts where the size is proportional to its contribution to the pooled effect size.

Genome-wide significant associations were found for DG1, DG6, DG7, DG9, DG11, as well as FUC-A (Table 1; Figure 1 and Figure 2). Association profiles for DG1, DG7, and DG9 are represented in their genomic context in Figure 1 for the associated region. Quantile–quantile plots for each association were consistent with an excess of true genetic associations, with modest genomic control inflation for each population (inflation factor <1.04 for all traits and each population as well as the meta-analysis), suggesting that the observed results were not due to population stratification (Figure 2A–2C).

**Table 1:** Genetic markers associated with plasma N-glycan levels.

Trait	Fucosylation status	Chr	SNP	Meta-analysis P-value	Meta-analysis sample size	Associated interval size, kb (no. of genes in interval)	Genes within associated interval	Major allele, minor allele (MAF)	Effect size for minor allele(s.e.) (z-score units)*
DG1	NF	14q23.3	rs7159888	3.46×10^−18^	2,441	650 (1)	FUT8	G, A (0.44)	0.26 (0.03)
DG6	CF	14q23.3	rs10483776	9.58×10^−09^	2,332	650 (1)	FUT8	A, G (0.21)	−0.22 (0.04)
DG7	AF	12q24.31	rs7953249	1.97×10^−08^	2,321	118 (3)	HNF1Α-C12orf43-OASL	A, G (0.45)	−0.17 (0.03)
DG7	AF	19p13.3	rs3760776	3.42×10^−12^	2,320	54 (3)	NRTN-FUT6-FUT3	G, A (0.11)	−0.34 (0.05)
DG9	AF, CF	19p13.3	rs3760776	3.51×10^−17^	2,333	54 (3)	NRTN-FUT6-FUT3	G, A (0.11)	−0.41 (0.05)
DG11	NF/CF, AF	12q24.31	rs735396	4.44×10^−08^	2,333	118 (3)	HNF1Α-C12orf43-OASL	A, G (0.38)	0.17 (0.03)
DG12	AF	19p13.3	rs3760776	9.44×10^−10^	2,330	54 (3)	NRTN-FUT6-FUT3	G, A (0.11)	−0.30 (0.05)
FUCA	AF	19p13.3	rs3760776	1.41×10^−12^	2,321	54 (3)	NRTN-FUT6-FUT3	G, A (0.11)	−0.34 (0.05)

*Effect size shown is the β-coefficient, which represents change in glycan levels measured in standard deviation units (after adjustment for covariates) per copy of the allele modelled. NF, non-fucosylated; CF, core fucosylated; AF, antennary fucosylated; See also Tables S1 and S2.

Fifteen SNPs located in the region encompassing the fucosyltransferase 8 gene (*FUT8*, Entrez GeneID: 2530) on chromosome 14 were significantly associated with plasma concentrations of desialylated glycan (DG) 1, the most significant being rs7159888 (p = 3.46×10^−18^) located 5′ of the gene. *FUT8* was also associated with DG6, however for this trait only one SNP, rs10483776, reached genome-wide significance (p = 9.58×10^−09^). All SNPs significantly associated with DG1 levels were in high LD (r^2^>0.5) and located between two recombination hotspots, while no associations were found with SNPs located outside these boundaries nor with other genes located within this association interval (Figure 1A). The effect size of the G allele of rs7159888 was −0.2617 (s.e. 0.0301) for DG1 in the meta-analysis of the 3 populations studied (standard deviation units, after adjustment for sex and age; Figure 2D). All significant SNPs in this region had a similar effect size (absolute value of the range: 0.1828–0.3251), accounting for between 1 and 6 percent of the trait variance after adjustment for age and sex. The effect of rs7159888 on DG1 was consistent across populations with similar amplitude and direction of effect (Figure 2D) with the effect for each population plotted separately along with the pooled effect. Haplotype analysis found that a single SNP model performed better than the 3- or 5-SNP haplotype model in every population.

A single SNP located on chromosome 19, rs3760776, was associated with DG7, DG9, DG12 and FUC-A (p = 3.42×10^−12^, p = 3.51×10^−17^, p = 9.44×10^−10^, p = 1.41×10^−12^). This SNP is located at the 5′ end of the fucosyltranferase 6 gene (*FUT6*, Entrez GeneID: 2528). The association interval for this SNP contains the *NRTN*, *FUT6* and *FUT3* genes (see Figure 1C), of which *FUT6* and *FUT3* are both biologically plausible candidates to explain the observed associations. The effect size of the G allele of rs3760776 is 0.3387 (s.e. 0.0487) for DG7 (standard deviation units, after adjustment for significant covariates: sex, age and fibrinogen); and 0.4104 (s.e. 0.0487), 0.2974 (s.e. 0.0486), and 0.3446 (s.e. 0.0486) for DG9, DG12 and FUC-A respectively (standard deviation units, after adjustment for age and fibrinogen). These effects account for 2% (DG7), 3% (DG9), 2% (DG12) and 2% (FUC-A) of the trait variance. A forest plot of the effect size of rs3760776 in each population and the meta for DG7 is presented in Figure 2F. Haplotype analysis suggested that a 5-SNP haplotype across this region has a stronger effect on these glycan levels than a single SNP model. Another fucosyltransferase gene (*FUT3*) is also within the region, so the causal variant(s) may affect one or both of these genes. The best 5-SNP haplotype contained rs3760776 and encompassed *FUT6* but not *FUT3* in every population and for every glycan group tested which suggests that the association is with *FUT6*, not *FUT3*.

The glycan structures which were significantly associated with genetic variants in the *FUT6* and *FUT8* genes are summarised in Table 1. Glycan group DG1 consists of a single structure GlcNAc_2_Man_3_GlcNAc_2_ that is known to be a substrate for the α1-6-fucosyltransferase (FUT8) (Table S1) [15], [33]. Group DG6 contains three glycan structures, two of which are core fucosylated so the results are consistent with the known biological role of FUT8. In contrast, groups DG7, DG9 and DG12 include glycans containing antennary fucose while FUC-A was derived as an overall measure of antennary fucosylation. *FUT6* encodes the enzyme fucosyltransferase VI which was reported to be the key enzyme responsible for the α_3_-fucosylation of plasma proteins [34]. The association of *FUT8* and *FUT6* genes with N-glycan structures containing core and antennary fucosylation is supported by their known biological functions [35] and the fact that they were identified in this study is an effective proof of principle that HPLC measured glycan levels can be used to identify genes that regulate protein glycosylation.

### Novel association of HNF1α with N-glycans

Two SNPs on chromosome 12, rs7953249 and rs735396, showed genome-wide significant associations with DG7 (p = 1.97×10^−08^, p = 1.75×10^−08^). The latter SNP was also associated with DG11 (p = 4.44×10^−08^), with an effect in the opposite direction, and was close to genome-wide significance with DG9 (Table S2). Both SNPs are located in the *HNF1α* (Entrez GeneID: 6927) gene region: rs7953249 is found 13 kb 5′ to the gene and rs735396 is in intron 9. Two other genes are found between the recombination hotspots that comprise the boundaries of the association interval, C12orf43 and OASL (Figure 1B). However, none of the most significantly associated SNPs are located in these genes and all SNPs with suggestive p-values (p<1×10^−05^) are located within *HNF1α* (Table S2). The effect size of the G allele of rs735396 is −0.1767 (standard deviation units, after adjustment for sex, age and fibrinogen; s.e. 0.0314) for DG7, which only contains glycans with antennary fucose, and in the opposite direction (0.1699 standard deviation units, after adjustment for age and fibrinogen; s.e. 0.0310) for DG11, which has no antennary fucose (Table 1). All significant SNPs in this region had a similar effect size (absolute value of the range: 0.1396–0.1767), representing 1–3% of the trait variance. Figure 2E shows the effect size for rs735396 with DG7 for each population separately and the pooled meta-analysis. Comparison of models including rs7953249 and rs735396 separately and combined suggests that the causal variant is located between these two SNPs. This was confirmed by analysis of imputed data based on HapMap release 2 with the most significant SNPs located across intron 1 of *HNF1α*.

### HNF1α regulates multiple stages in protein fucosylation: (1) regulation of GDP-fucose biosynthesis

**Figure 3:**
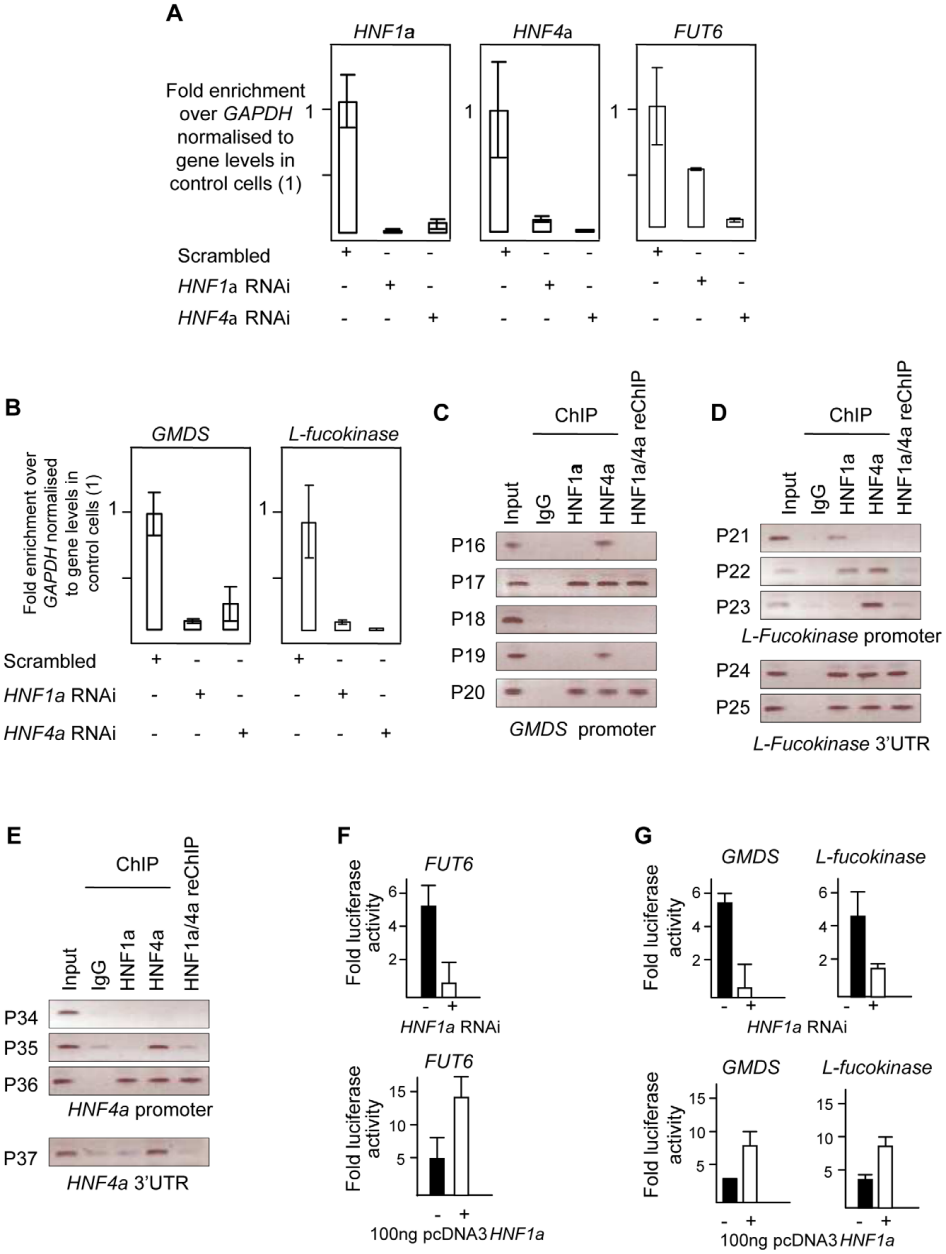
HNF1α is major regulator of fucose synthesis pathway genes. A). Real time PCR for *HNF1*α, *HNF4α* and *FUT6* RNAs after *HNF1α* and *HNF4α* RNA interference (RNAi) in HepG2 cells. B). Real time PCR for *GMDS* and *L-Fucokinase* transcripts after *HNF1α* and *HNF4α* RNA interference (RNAi) in HepG2 cells. Expression levels were compared to *GAPDH* transcripts. All experiments were performed in triplicate. C). Chromatin immunoprecipitation (ChIP) analysis of regions as determined by the indicated primer sets for the *GMDS* promoter. D). ChIP analysis of regions as determined by the indicated primer sets for the *L-Fucokinase* promoter and 3′ UTRs. E). ChIP analysis of regions as determined by the indicated primer sets for the *HNF4α* promoter and 3′ UTRs. F). Reporter activity of the control *FUT6* promoter upon *HNF1α* RNAi in HepG2 cells or pcDNA3-HNF1α expression in HEK293 cells. G). Reporter activity of the *GMDS* and *L-Fucokinase* promoters upon *HNF1α* RNAi in HepG2 cells or pcDNA3-HNF1α expression in HEK293 cells. Abbreviations: HNF: Hepatocyte nuclear factor; RNAi: RNA interference, GMDS: GDP-mannose 4,6-dehydratase; L-Fuc: L-Fucokinase; FUT: Fucosyltranferase. 3′UTR: 3′ un-translated region. reChIP: re-precipitation (or sequential) ChIP. See also Figure S2 for gene expression results in PANC1 cell line.

The shared characteristic of all glycan groups that showed association with *HNF1α* SNPs was the presence or absence of antennary fucose (Table S2). We hypothesised that HNF1α transcriptionally regulates the expression of genes involved in the separates steps of fucosylation. This is supported by the fact that a functionally related transcription factor, HNF4α was previously shown to bind the regulatory elements of the GDP-mannose-4,6-dehydratase (GMDS) gene in a genome-wide ChIP-ChIP. GMDS is involved in the *de novo* pathway of L-fucose synthesis to produce GDP-fucose, the substrate used by both core and antennary fucosyltransferases to N-glycosylated proteins [24]. Moreover, HNF4α directly regulates the expression of the hepatic fucosyltransferase VI gene (*FUT6*) [36]. Therefore, we tested whether HNF1α and/or HNF4α might regulate other genes involved in GDP-fucose biosynthesis. To this end, *HNF1α* and *HNF4α* were transiently knocked-down in liver and pancreatic cell lines using RNA interference. Both *HNF1α* and *HNF4α* expression levels decreased upon knockdown of either of them in hepatocytes (Figure 3A). In pancreatic cells, *HNF1α* knockdown up-regulates *HNF4α* expression but the reverse is not true (Figure S1). This confirms the differential regulation of gene expression downstream of HNFs in liver *vis-a-vis* pancreas [28], [37]. It also corroborated recent findings in murine *Hnf1α* hetrozygote pancreas, where the levels of *Hnf4α* mRNA increase [38].

As a positive control, the expression of *FUT6*, a known target of HNF4α in hepatocytes, was first analysed. The ablation of the *HNF4α* transcript abolished the expression of *FUT6* in HepG2 cells confirming that the knockdown was effective. Surprisingly, knockdown of HNF1*α* resulted in 50% reduction in *FUT6* transcript levels suggesting that HNF1*α* also regulate FUT6 expression in HepG2. This experiment suggested that our hypothesis may potentially explain and provide a direct link between HNF1α and the fucosylation genes. Therefore, we focused on the genes responsible for fucose biosynthesis, a rate limiting step in protein fucosylation. To this end, we analysed the expression of *GMDS* and *L-Fucokinase* which regulate *de novo* and salvage pathways of fucose synthesis, respectively. In HepG2 liver cells, *HNF1α* and *HNF4α* knockdown resulted in dramatic down-regulation in the expression of *GMDS* (91 and 77%, respectively) and *L-Fucokinase* (92 and 98%, respectively) (Figure 3B). In the pancreatic Panc1 cell lines, *HNF4α* RNAi resulted in a 70% decrease in *GMDS* and *L-Fucokinase* transcript levels (Figure 1). However, *HNF1α* RNAi led to a 90% reduction in *GMDS* transcript levels but did not affect *L-Fucokinase* mRNA abundance (Figure 1). This suggests that HNF1α regulates *de novo* synthesis of d-fucose in both cell lines tested (liver and pancreas), but only the salvage pathway in the liver cell line tested. HNF4α, on the other hand, regulates both pathways in both cell types tested.

We therefore focused on HNF1α direct transcriptional regulation of *HNF4α*, *GMDS* and L-*Fucokinase* in HepG2 cells. In order to investigate the latter, we performed a bioinformatics analysis to delineate *in silico* HNF1α and HNF4α binding sites. First, we assessed the conservation of regulatory elements (at the 5′ and 3′ end) between human and other primates as described previously [39]. It was recently shown the sites are not conserved between primates and rodents [40]. Second, the conserved regions were then mined for potential sites using ECR browser and the TRANSFAC database [41]. Finally, the potential sites were analysed manually to ascertain the likely binding sites based on homology to HNF1α and HNF4α consensus binding sites mined using genome-wide ChIP analyses [40], [42]. This limited our analysis to 5 sites (primer pairs P16 to P20, Figure 3C) in the *GMDS* promoter, 3 sites in the promoter (primer pairs P21 to P23, Figure 3D) as well as 2 sites at the 3′end (primer pairs P24 and P25, Figure 3D) of the *L-Fucokinase* gene and 3 sites in the promoter (primer pairs P34 to P36, Figure 3D) as well as a single site at the 3′ end (primer pair P37) of the *HNF4α* gene. The primer pairs are less than 1Kbps away from each other and some contained both HNF1α and HNF4α binding sites (or half sites) within the 200bps amplifiable regions.

Using these primer pairs, we performed chromatin immunoprecipitation (ChIP) assays to delineate the occupancy of these sites by HNF1α, HNF4α or both proteins as described earlier [43], [44].

In HepG2, both HNF1α and HNF4α bind the promoters of *GMDS* (P17, Figure 3C), L-*Fucokinase* (P22 although the two factors cannot be re-precipitated, Figure 3D) and *HNF4α* (P36, Figure 3E). Also, we show binding of HNF1α and HNF4α at the 3′UTR of L-*Fucokinase* as well as HNF4α binds the 3′UTR of *HNF4α* (Figure 3D and 3E, respectively). The interactions of these proteins is not affected by shearing as the primers acts as genomic controls for each other and no signal above background was apparent in the IgG isotype control antibody. Together, the data suggests a complex network of interactions between HNF4α and HNF1α to regulate fucose biosynthesis gene expression and point to a novel and an unappreciated role for HNF1α in regulating the two genes studied (*GMDS* and L-*fucokinase*). We further investigated the role of HNF1α in regulating the activity of the promoter regions bound by HNF factors (i.e. regions amplified by primer pairs P17, P22 and P37). We cloned these fragments into luciferase expressing vector (Promega's pGL4-basic) and assayed for reporter activity in two systems to delineate whether HNF1α is necessary to drive reporter expression (RNAi in HepG2 cells) and sufficient (expression of HNF1α in HEK293 cells that do not express endogenous HNF1α). Knockdown of *HNF1α* leads to a downregulation in the activity of both *GMDS* (5 fold reduction) and L-*Fucokinase* (2 fold reduction) promoter regions. Conversely, HNF1*α* overexpression leads to the induction of the luciferase activity in reporters driven by the two promoter regions. Put together, the expression data combined with the ChIP analysis and the reporter activity results strongly support a direct role for HNF1α in regulating the two key genes *GMDS* and *L-Fucokinase* that are responsible for *de novo* and salvage pathway of fucose synthesis.

### HNF1α regulates multiple stages in protein fucosylation: (2) transcriptional regulation of core and antennary fucosyltransferases

**Figure 4:**
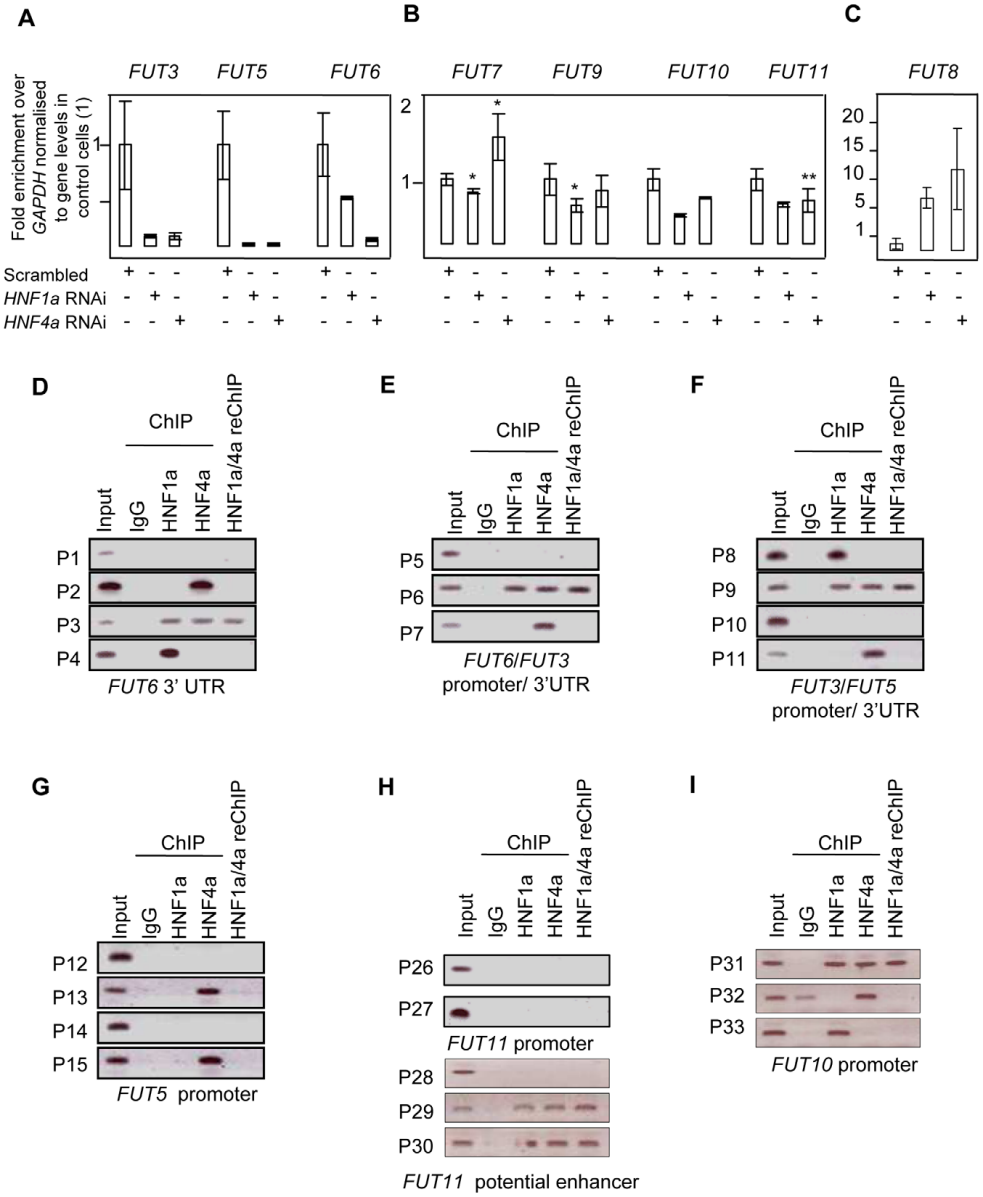
HNF1α is major regulator of the fucosyltransferase genes. Real time PCR for *FUT3*, *FUT5* and *FUT6* RNAs (A); *FUT7*, *FUT9*, *FUT10* and *FUT11* (B); *FUT8* (C) after *HNF1α* and *HNF4α* RNA interference (RNAi) in HepG2 cells. H) analyses show that Hnf1α (HNF1) and Hnf4α (HNF4) bind predicted regulatory elements for key fucosylation genes in liver cells. D), E), F), G), H) and I) Binding of HNF1α and HNF4α to predicted regulatory elements of the *FUT3*, *FUT5, FUT6, FUT10* and *FUT11.* Abbreviations: the gene names are detailed in Figure 1; P1–P37: Primer sets 1 to 37; 3′UTR: 3′ un-translated region. reChIP: re-precipitation (or sequential) ChIP.

After confirming the role of HNF1α in the biosynthesis of GDP-fucose, we analysed the role of HNF1α and HNF4α in the regulation of the expression of fucosyltransferase (FUT) genes (FUT3-11) in HepG2 and Panc1 cell lines to assess whether these hepatic factors regulate other stages of protein fucosylation. In HepG2 cells, *HNF1*α knockdown down-regulated the expression of all *FUT* genes (Figure 4A and 4B), except *FUT8* whish was induced upon the loss of HNF1α (Figure 4C). *HNF4α* knockdown led to a statistically significant downregulation of *FUT3*, *FUT5*, *FUT6*, *FUT10*, *FUT11* but not *FUT7* or *FUT9* (Figure 4A and 4B). Conversely, *FUT8* expression levels increased 10 fold upon the loss of *HNF4α* ((Figure 4C). *FUT4* was not expressed in HepG2 cells confirming earlier studies [45]. In the pancreas, all *FUT* genes were down-regulated (Figure 2) pointing to a key role for HNF1α in the regulation of fucosylation in the pancreas. Knockdown of *HNF4α* in liver cells reduced the expression of all *FUT* genes analysed except *FUT7* or *FUT9,* but to a lesser extent than *HNF1α* knockdown (Figure 4A and 4B), however, *FUT8* was again up-regulated (Figure 4A and 4B). The data supports a wider effect of HNF1α on the expression of the 8 fucosyltransferase genes compared to HNF4α. The data also suggests that HNF1α and HNF4α downregulate *FUT8*, which adds fucose to the core glycan, in contrast to all other FUTs that add fucose to the antennary arms of glycans [33]. We observed a rather high correlation between concentrations of antennary and core fucose in our population samples (r = 0.574, p = 4.01×10^−85^), indicating that the availability of the common substrate of both core and antennary FUTs, GDP-fucose, is a rate-limiting factor in protein fucosylation. It therefore appears that HNF1α not only enhances the activity of antennary FUTs but also, by down-regulating FUT8, increases the amount of GDP-fucose available for antennary fucosylation.

*FUT3, FUT6* and *FUT5* were the only FUTs to be highly repressed (more than 3-fold) upon the loss of both HNF1α and HNF4α in liver cells (Figure 4A), suggesting a co-regulation of the three genes. In pancreatic cells, *FUT3* and *FUT6*, but not *FUT5* followed the same dynamics (Figure 2). *FUT3* and *FUT6* expression was not repressed upon *HNF4α* loss (Figure 2). This could be explained by a differential role for HNF4α in regulating *FUT5* but not *FUT3* or *FUT6*. These data suggest that HNF1α is the major regulator of the fucosylation pathway in both liver and pancreatic cell lines. While HNF4α also regulates the expression of these genes, its role is probably secondary to HNF1α. However, none of the genes studied here have previously been shown to be regulated *in vivo* by HNFs. Only the *GMDS* promoter has previously been shown to be chromatin immunoprecipitated with HNF4α antibody [28].

Bioinformatic analysis showed that *FUT3*, *FUT5* and *FUT6* are clustered in one locus in the human genome (see and Figure S3) [39]. This also corroborated our findings that *FUT3*, *FUT5* and *FUT6* are co-regulated downstream of HNF4α and HNF1α (Figure 4A). However, the *FUT3/5/6* cluster was neither syntenic nor conserved in the mouse genome. We therefore focused on primate conservation only.

The promoter, intergenic and 3′ regulatory element conserved regions were analysed for HNF binding sites as detailed above for *GMDS* and *L-Fucokinase*. This analysis identified a limited number of sites in regulatory regions of *FUT3*, *FUT5*, *FUT6*, and *FUT10*. It did not identify any binding sites *in silico* in the *FUT11* promoter, but a highly conserved long range enhancer was found within the *ADK* gene, that is 650 kb upstream and rich in HNF binding sites. We were unable to detect any HNF binding sequences within the *FUT8* regulatory elements analysed.

Using ChIP, the binding of HNF1α and HNF4α to the putative response elements identified *in silico* was analysed. ChIP analysis showed that HNF1α and HNF4α bound multiple sequences within the predicted regulatory regions of multiple FUT genes, including *FUT3*, *FUT5*, *FUT6*, *FUT10*, *FUT11* (Figure 4D–4I). HNF4α, and not HNF1α, bound the promoter of *FUT5* (P13 and P15, Figure 4G). The unique binding of HNF4α to the promoter of *FUT5* corroborated our findings that knockdown of HNF4α in pancreatic PANC1cells abolished the expression of *FUT5* but not *FUT3* or *FUT6* (Figure 2).

Using re-precipitation (reChIP), we confirmed that both HNF transcription factors bound (i) the promoters of *FUT3*, *FUT6* and *FUT10* (Figure 4E, 4F, 4D, and 4I respectively); (ii) 3′UTRs of *FUT6* (Figure 4D); and (iii) the long range enhancer 650 kb upstream of *FUT11* (Figure 4H). This shows that HNF1α and HNF4α are potential regulators of the expression of these genes *in vivo.*

## Discussion

**Figure 5:**
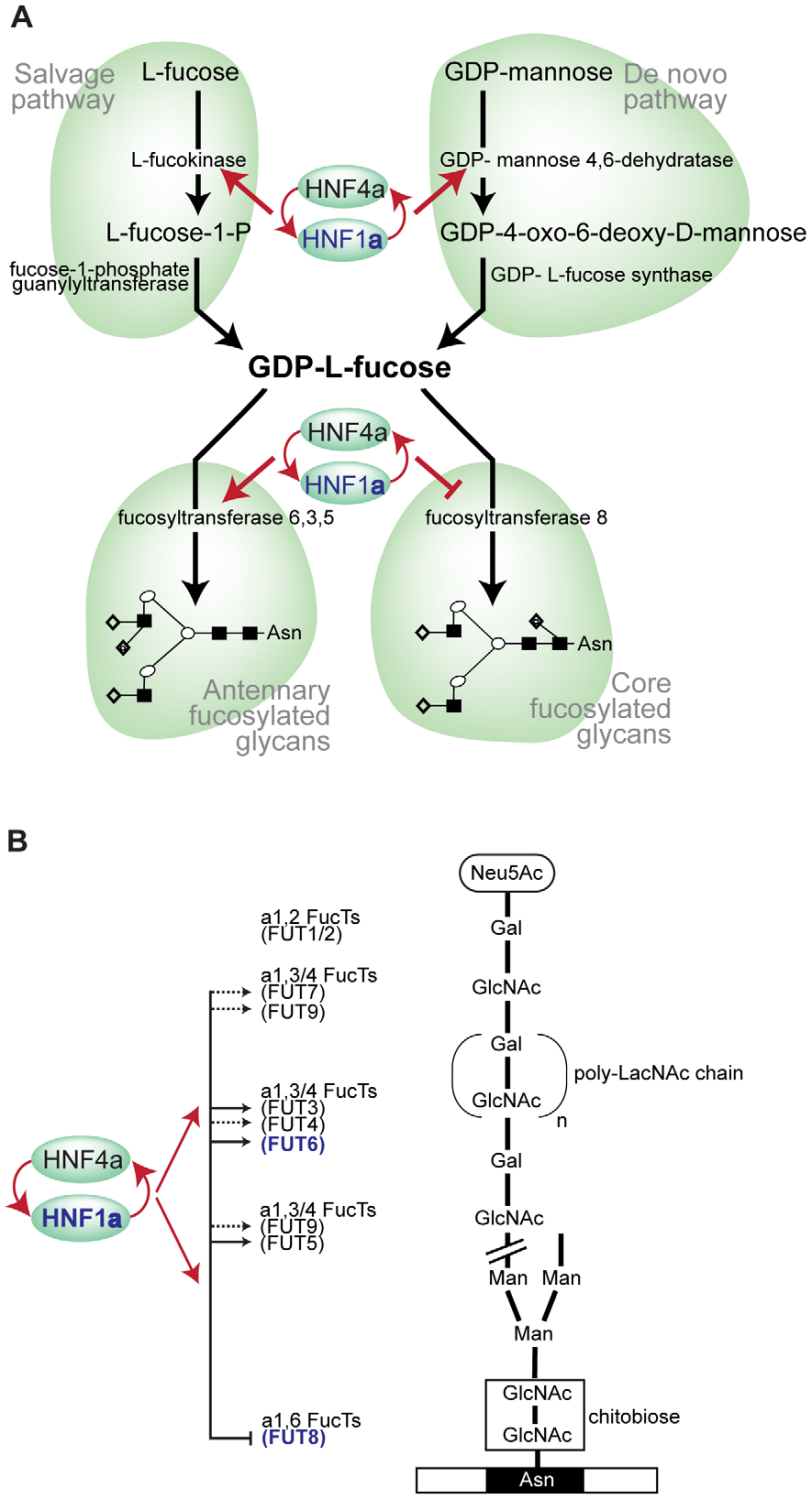
HNF1α is at the heart of fucosylation regulation. A). Diagram showing HNF1α and HNF4α regulation of pathways involved in fucose synthesis. B). Pathways of core (tri-mannosyl-chitobiose) and antennary fucosylation and their proposed regulation by the transcription factors Hnf1α and Hnf4α, encoded by the *HNF1α* and *HNF4α* genes respectively. Red arrows indicate genes that are targets of Hnf1α/Hnf4α. Solid lines show direct regulation and dashed lines show indirect regulation of the FUT genes shown. Genes shown in blue depict GWAS identified genes (*HNF1α*, *FUT6* and *FUT8*). Abbreviations: Neu5Ac, N-Acetylneuraminic (sialic) acid; GlcNAc, N-acetylglucosamine; Man, mannose; Gal, galactose; poly-LacNAc, Galβ1,4GlcNAc (LacNAc) antennary chains; Asn, asparagine residue on glycosylated protein.

By performing the first genome-wide association analysis (GWAS) of protein glycosylation we have taken the first steps towards the mapping of the complex network of genes that regulate protein N-glycosylation. We also identified common variants in three genes which exert a relatively strong influence on N-glycans in plasma (1–6% of variance explained). Importantly, all of the identified genes (*FUT6, FUT8 and HNF1α)* are involved in fucosylation, indicating that the addition of this unusual sugar may be a rate-limiting step in N-glycan synthesis. A gene encoding the transcription factor HNF1α, with previously unknown biological links to glycosylation, is shown to be strongly associated with the relative proportions of plasma N-glycans. The possible function(s) of HNF1α are a focus of intense current interest following its recently reported associations in GWAS with plasma C-reactive protein (CRP) [27], gamma-glutamyl transferase (GGT) [28], LDL cholesterol and apolipoprotein [29], [31] and coronary artery disease [29], [46]. Our analysis of gene knockdowns (RNAi) showed that HNF1α is an upstream regulator of several key genes involved in different stages of the fucosylation pathway. We have demonstrated that HNF1α binds the promoters *in vivo*, and is necessary and sufficient for the *in vitro* expression, of two genes, fucokinase and *GMDS*, required for *de novo* and salvage pathways of fucose synthesis, respectively (Figure 5C). Fucose synthesis is the rate limiting step for fucosylation in eukaryotes and prokaryotes [35] and, by up-regulating its synthesis, HNF1α increases the availability of fucose to the glycosylation machinery. In addition, HNF1α directly regulates the expression of several fucosyltransferase (FUT) genes (Figure 5D). Our results also demonstrate that HNF1α reciprocally regulates core *versus* antennary fucosylation; while activating FUTs involved in antennary fucosylation, HNF1α represses *FUT8*, which adds fucose to the core-GlcNAc. In this way, HNF1α decreases the consumption of GDP-fucose for core-fucosylation, and further increases the pool of fucose available for antennary fucosylation.

Having shown this novel regulation of fucosylation genes, we scanned earlier genome wide studies for HNF factors to identify whether these genes were picked up. In fact, other genome wide studies support our findings. Boyd et al (2009) mapped HNF4α binding to both FUT2 and FUT5 in intestinal epithelial cells [42]. A genome wide prediction study for HNF4α functional binding sites identified FUT6, FUT5, FUT9, GMDS and FUT12 as functional targets [47].

We hypothesize that the role of HNF1α and its transcriptional co-factor HNF4α in the regulation of fucosylation is an essential part of mounting an acute phase response to infection in humans. Antennary fucosylation of their glycoprotein ligands is needed for binding of E-, L- and P-selectins to their target cells and the initiation of inflammation [48]. The decrease in fucosylation in the rare Leukocyte Adhesion Deficiency II (LAD II) impairs neutrophil function, which can be restored by oral administration of fucose [49]. Recently, we have reported moderate correlations between fucosylated plasma N-glycans and components of the acute phase response [15], which are also highly glycosylated and have high content of antennary-fucose [50]. Mounting a successful acute-phase response requires a rapid increase in the concentration of acute-phase proteins and this in turn is dependent on their efficient fucosylation. Our results indicate that fucosylation is a rate-limiting step in plasma protein glycosylation, and by both increasing *de novo* and salvage synthesis of GDP-fucose, up-regulation of antennary fucosyltransferases and down-regulation of core-fucosyltransferase, HNF1α appears to be a master regulator of this process. Variants in *HNF1α* and *HNF4α* genes were previously reported to be associated with concentrations of acute phase proteins in human plasma [24], [27]. Plasma protein fucosylation plays an important role in inflammation [22] and the central role of HNF1α in the regulation of multiple genes involved in fucosylation may be the molecular mechanism behind the reported association between common variants in HNF1α and inflammatory markers (such as CRP) as well as several diseases in which inflammation plays a key pathogenic role (such as coronary artery disease, inflammatory bowel disease and cancer).

## Materials and Methods

### Study populations and genotyping

All three populations recruited adult individuals within a community irrespective of any specific phenotype. The CROATIA-VIS and CROATIA-KORCULA studies are both cohorts from the Croatian Dalmatian islands recruited in 2003–2004 and 2007 respectively. The ORCADES study is ongoing with participants recruited from the Orkney islands in Scotland. Fasting blood samples were collected, biochemical and physiological measurements taken and questionnaires of medical history as well as lifestyle and environmental exposures collected following similar protocols.

The CROATIA-VIS study includes 1008 Croatians, aged 18–93 years, who were recruited from the villages of Vis and Komiza on the Dalmatian island of Vis during 2003 and 2004 within a larger genetic epidemiology program [32].

The CROATIA-KORCULA study includes 969 Croatians between the ages of 18 and 98 [32]. The field work was performed in 2007 in the eastern part of the island, targeting healthy volunteers from the town of Korčula and the villages of Lumbarda, Žrnovo and Račišće.

The Orkney Complex Disease Study (ORCADES) is an ongoing study in the isolated Scottish archipelago of Orkney [32]. Data for participants aged 18 to 100 years, from a subgroup of ten islands, were used for this analysis.

DNA samples were genotyped according to the manufacturer's instructions on Illumina Infinium SNP bead microarrays (HumanHap300v1 for the CROATIA-VIS cohort, HumanHap300v2 for the ORCADES cohort and HumanCNV370v1 for the CROATIA-KORCULA cohort). Genotypes were determined using Illumina BeadStudio software. Genotyping was successfully completed on 991 individuals from CROATIA-VIS, 953 from CROATIA-KORCULA and 761 from ORCADES.

### Ethics statement

All studies conformed to the ethical guidelines of the 1975 Declaration of Helsinki and were approved by appropriate ethics boards with all respondents signing informed consent prior to participation.

### Glycan release and labelling

The N-glycans from plasma sample (5 µl) proteins were released and labelled with 2-aminobenzamide (LudgerTag 2-AB labelling kit Ludger Ltd., Abingdon, UK) as described previously [13]. Labelled glycans were dried in a vacuum centrifuge and redissolved in known volume of water for further analysis.

### Sialidase digestion

After initial HPLC quantification sialidase digestion was performed to improve measurement precision. Aliquots of the 2-AB-labeled glycan pool were dried down in 200-µl microcentrifuge tubes. To these, the following was added: 1 µl of 500 mM sodium acetate incubation buffer (pH 5.5), 1 µl (0.005 units) of ABS, *Arthrobacter ureafaciens* sialidase (releases α2–3, 6, 8 sialic acid, Prozyme) and H_2_O to make up to 10 µl. This was incubated overnight (16–18 h) at 37°C and then passed through a Micropure-EZ enzyme remover (Millipore, Billerica, MA, USA) before applying to the HPLC.

### Hydrophilic interaction high-performance liquid chromatography (HILIC)

Released glycans were subjected to hydrophilic interaction high performance liquid chromatography (HILIC) on a 250×4.6 mm i.d. 5 µm particle packed TSKgel Amide 80 column (Tosoh Bioscience, Stuttgart, Germany) at 30°C with 50 mM formic acid adjusted to pH 4.4 with ammonia solution as solvent A and acetonitrile as solvent B. 60 min runs were on a 2795 Alliance separations module (Waters, Milford, MA). HPLCs were equipped with a Waters temperature control module and a Waters 2475 fluorescence detector set with excitation and emission wavelengths of 330 and 420 nm, respectively. The system was calibrated using an external standard of hydrolyzed and 2-AB-labeled glucose oligomerase from which the retention times for the individual glycans were converted to glucose units (GU) [51]. Glycans were analyzed on the basis of their elution positions and measured in glucose units then compared to reference values in NIBRT's “GlycoBase v3.0 ” database available at http://glycobase.nibrt.ie) for structure assignment [52].

HPLC analysis was performed partly in the National Institute for Biotechnology and Training (NIBRT) in Dublin, Ireland, and partly in the Glycobiology laboratory of Genos Ltd in Zagreb, Croatia. Both laboratories used the same columns and separation conditions. Duplicate analysis of a number of samples was performed and confirmed full reproducibility of the analytical results both within and between laboratories.

### Glycan structural features

Levels of glycans sharing the same structural features were approximated by adding the structures having same characteristics: Core fucosylated glycans (FUC-C)  =  DG6/(DG5+DG6)*100; Antennary fucosylated glycans (FUC-A)  =  DG7/(DG5+DG7)*100.

### Genotype and phenotype quality control

Genotyping quality control was performed using the same procedures for all cohorts. Individuals with a call rate less than 97% were removed as well as SNPs with a call rate less than 98% (95% for CROATIA-VIS), minor allele frequency less than 0.02% or Hardy-Weinburg equilibrium p-value less than 1×10^−10^. Differences in SNP call rate threshold were used to account for observed differences between genotyping arrays. 924 individuals passed all quality control thresholds from CROATIA-VIS, 898 from CROATIA-KORCULA and 737 from ORCADES.

Extreme outliers were removed for each glycan measure to account for errors in quantification and to remove individuals not representative of normal variation within the population. An individual was classified to be an extreme outlier if their measure for the trait was more than 3 interquartile distances away from the mean.

### Genome-wide association analysis

Each trait was tested for normality within each cohort then the transformation that performed best for all cohorts was used. Models including sex, age and fibrinogen as covariates were tested for each cohort separately. Any covariate that was significant within any cohort was included as a covariate in the final model.

Genome-wide associations were performed for all glycan measures using the same transformation to normality and covariates for each cohort separately then combined in a meta-analysis. The “mmscore” function of the GenABEL package for R statistical software [53] was used for the association test under an additive model. This score test for family based association takes into account pedigree structure and allowed unbiased estimations of SNP allelic effect when relatedness is present between examinees [54]. The relationship matrix used in this analysis was generated by the “ibs” function of GenABEL which used IBS genotype sharing to determine the realised pairwise kinship coefficient. Meta-analysis was performed using the MetABEL package for R [53]. An association was considered statistically significant at the genome-wide level if the p-value for an individual SNP was less that 5×10^−8^ (based on Bonferroni correction to account for multiple testing). All identified SNPs that reached significance or seemed to be suggestive of significance were visualised using Haploview software [55].

### Association interval

An associated interval for a region of interest was defined by determining the HapMap SNPs in linkage disequilibrium of *r* ^2^ >0.5 with the most significantly associated SNP in the region using the web-based program SNAP [56]. The bounds of the associated interval were determined by the flanking HapMap recombination hotspots.

### Haplotype analysis

Haplotype analysis was performed on “unrelated” individuals in each population separately to account for possible allele frequency and haplotype differences between populations. Individuals were considered to be unrelated with a kinship coefficient of less than 0.05 (first cousins once removed). This left 525 individuals in the CROATIA-VIS cohort, 568 in CROATIA-KORCULA and 263 in ORCADES. An EM based algorithm was used to infer haplotypes from genotypic data. The “scan.haplo” function of the GenABEL package for R [53], which calls the “haplo.score.slide” function of the haplo.stats package for R [57], was used to test a sliding window of 3- and 5-SNP haplotypes across the associated interval. These results were compared to a single SNP model across the same region obtained using the “qtscore” function of the GenABEL package for R. A significant difference between haplotype and single-SNP analysis was determined using the Akaike information criterion [58].

### ChIP identification of HNF binding sites in FUT genes

To establish whether HNF1α and HNF4α bind the regulatory elements of the fucosylation genes, their genomic loci were analysed using bioinformatics to identify HNF response elements. Conserved elements between human and mouse genomes [39] were analysed initially to delineate the binding sites of HNF1α and HNF4α using the TRANSFAC database and the ECR browser (http://ecrbrowser.dcode.org/). Primers for ChIP, reChIP and real-time PCR are listed in Text S1.

### RNA interference

Production of the RNA duplexes for RNA interference was described in details earlier (Kittler et al., 2005). The target sequences (see Text S1) against *HNF1α* and *HNF4α* were designed using the siDESIGN Center (Dharmacon). The Trasnfection of HepG2 and PANC1 cells was carried out as described by (Yu et al., 2002).

### Luciferase assays

The PCR products of ChIP primers (Sequences are detailed in the Text S1) were cloned into pGEM-T easy vector (Promega) and subcloned into pGL4 vectors (Promega) as described earlier (Essafi et al., 2005). pGL4-*luc* constructs (100 ng) and internal control of pRLTK (20 ng) renilla plasmid were transiently co-transfected into HepG2 and PANC1 cells (10^5^) using the calcium phosphate co-precipitation. Cells were harvested 48 hr post-transfection for luciferase reporter assay using the Dual-Luciferase reporter assay system (Promega). The luciferase activity was normalized by *Renilla* luciferase activity. All assays were performed in three separate experiments done in triplicate.

### ChIP

ChIP was carried out on HepG2 cells essentially as detailed earlier (Essafi et al., 2005). The antibodies used were HNF1α (sc-6547) and HNF4α (sc-6556) from Santa Cruz Biotechnology. The corresponding control IgG antibodies were from Sigma-Aldrich.

### Real-time PCR

RNA isolation, cDNA synthesis and Real time PCR were performed as described earlier (Birkenkamp et al., 2007). PCR primer sequences are listed in Text S1.

## Supporting Information

Figure S1 PANC1 cells were treated with RNAi against *HNF1α* and *HNF4α*. The expression levels of the indicated genes were analysed by real time SYBR Green PCR.
(0.28 MB TIF)

Figure S2 PANC1 cells were treated with RNAi against *HNF1α* and *HNF4α*. The expression levels of the indicated genes were analysed by real time SYBR Green PCR.
(0.41 MB TIF)

Figure S3 The clustering of FUT3, FUT5 and FUT6 on chromosome 19. The position of primer pairs used for ChIP are indicated at the bottom of the figure.
(0.39 MB TIF)

Table S1 (A) Main structures present in glycan groups for which Genome-wide significant associations were found (SNPs in *FUT8*, *FUT6* and *HNF1*α genes). (B) Glycan structures present in different HPLC peaks.
(0.10 MB DOC)

Table S2 Desialylated glycan traits (DG1-13, FUC-A, FUC-C) and their associations with *FUT6*, *FUT8* and *HNF1α* SNPs showing their effect sizes (Beta) in standard deviation units (with standard errors) and p-values. The fucosylation status of each trait is also shown.
(0.06 MB DOC)

Text S1 Supplemental Materials and Methods.
(0.05 MB DOC)
