# Supplementary figures and images for: Genomics Meets Glycomics—The First GWAS Study of Human N-Glycome Identifies HNF1α as a Master Regulator of Plasma Protein Fucosylation
Source: PLoS Genet. 2010 Dec 23;6(12):e1001256. doi: 10.1371/journal.pgen.1001256 (PMC3009678; doi:10.1371/journal.pgen.1001256)

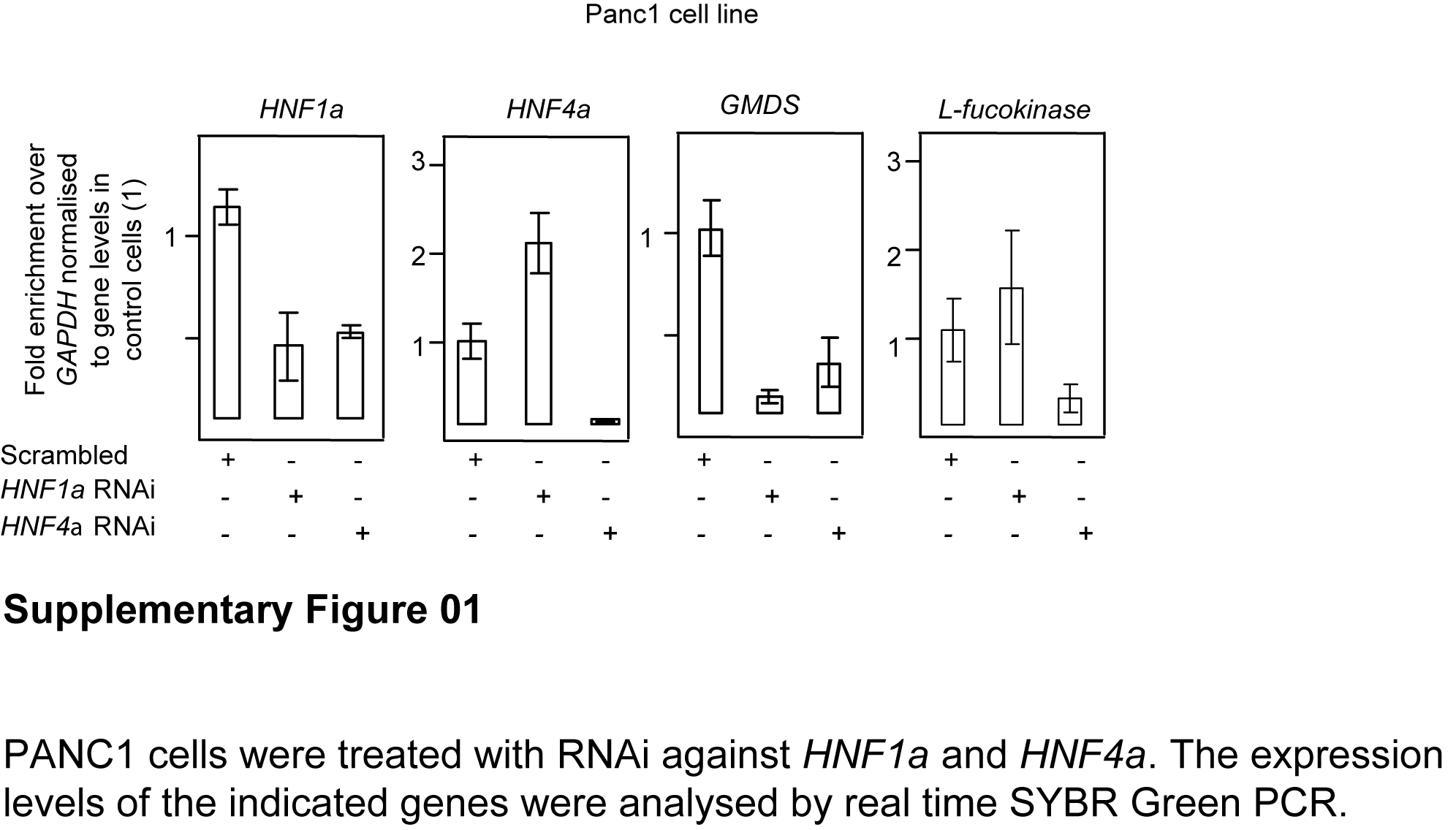

Supplement: Figure S1 — PANC1 cells were treated with RNAi against HNF1α and HNF4α. The expression levels of the indicated genes were analysed by real time SYBR Green PCR. (0.28 MB TIF) [file pgen.1001256.s001.tif]

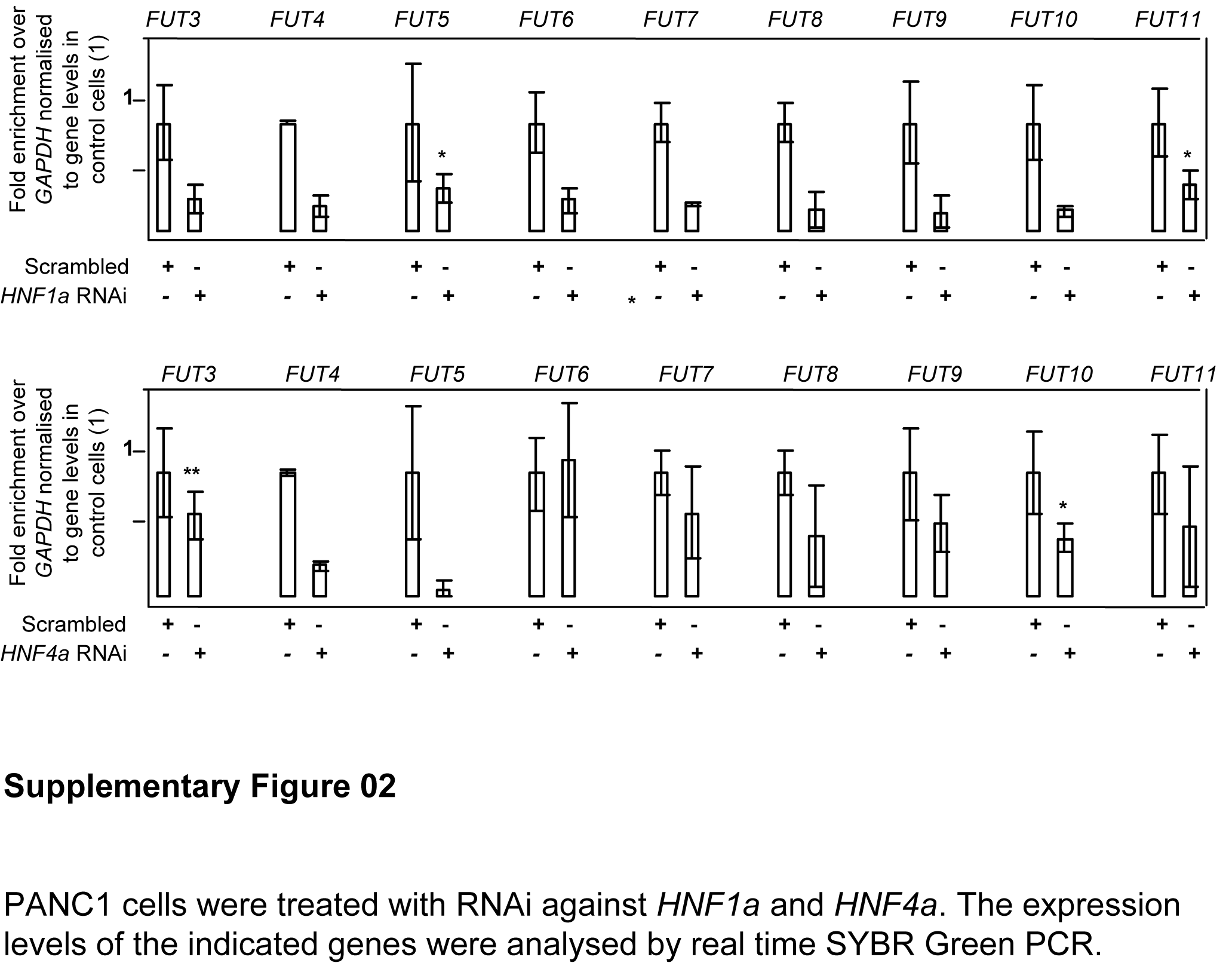

Supplement: Figure S2 — PANC1 cells were treated with RNAi against HNF1α and HNF4α. The expression levels of the indicated genes were analysed by real time SYBR Green PCR. (0.41 MB TIF) [file pgen.1001256.s002.tif]

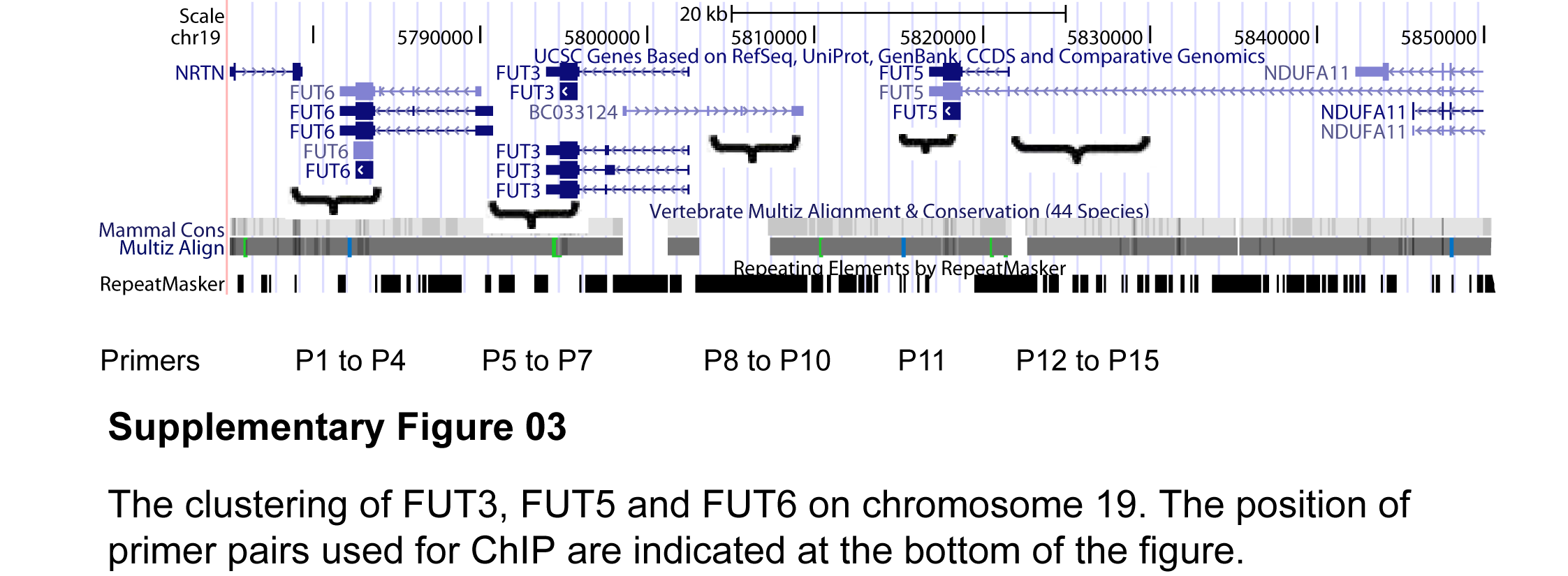

Supplement: Figure S3 — The clustering of FUT3, FUT5 and FUT6 on chromosome 19. The position of primer pairs used for ChIP are indicated at the bottom of the figure. (0.39 MB TIF) [file pgen.1001256.s003.tif]
