# Supplementary material for: Genomics Meets Glycomics—The First GWAS Study of Human N-Glycome Identifies HNF1α as a Master Regulator of Plasma Protein Fucosylation
Source: PLoS Genet. 2010 Dec 23;6(12):e1001256. doi: 10.1371/journal.pgen.1001256 (PMC3009678; doi:10.1371/journal.pgen.1001256)
Supplement: Table S1 — (A) Main structures present in glycan groups for which Genome-wide significant associations were found (SNPs in FUT8, FUT6 and HNF1α genes). (B) Glycan structures present in different HPLC peaks. (0.10 MB DOC) [file pgen.1001256.s004.doc]

**Table S1a**

| **Gene**  (chromosome) | **Peak** | **Main complex glycan structures present** |
| --- | --- | --- |
| FUT8  (Chromosome 14) | DG1 | **2AB**  A2 |
|  | D­­­G6 | FA2G2  **2AB**  FA2BG2  **2AB** |
| FUT6  (Chromosome 19) | DG7 | A2F1G2  **2AB** |
|  | DG9 | A3F1G3  **2AB** |
|  | DG12 | A4F1G4  **2AB** |
|  | FUC-A | represents biantennary structures with fucose on antenna |
| HNF1Α  (Chromosome 12) | DG7 | A2F1G2  **2AB** |
|  | DG11 | A4G4  **2AB**  FA4G4  **2AB** |

Glycan structures are presented schematically to denote the construction of the structure.

- N-acetylglucosamine, - mannose, - galactose, - fucose; 2AB – 2-aminobenzamide

Table S1b

| **Peak** | **Structure** |
| --- | --- |
| DG1 | A2 |
| DG2 | FA2  A2B  A1G1 |
| DG3 | M5  A2[6]G1  A2[6]BG1 FA2B |
|
|
| DG4 | FA2[6]BG1  FA2[3]G1  FA2[3]BG1  M4A1G1  A2[3]G1  A2[3]BG1 |
|
| DG5 | A2G2  A2BG2  M6D1, D2  M6D3 |
|
| DG6 | FA2G2  M5A1G1  FA2BG2 |
|
| DG7 | A2F1G2  M7D3  M7D1 |
| DG8 | A3G3  FA3G3 A2F2G2  M8D2, D3  M8D1,D3 |
|
|
|
| DG9 | A3F1G3 FA3BG3 |
| DG10 | FA3F1G3 M9 |
| DG11 | A4G4  A4BG4  FA4G4 A3F2G3 |
| DG12 | A4F1G4 |
| DG13 | A4F2G4 A4G4Lac  FA4F1G4 |

Structure abbreviations: all N-glycans have two core GlcNAcs; F at the start of the abbreviation indicates a core fucose α1-6 linked to the inner GlcNAc; M*x*, number (*x*) of mannose on core GlcNAcs; D1 indicates that the α1-2 mannose is on the Manα1-6Manα1-6 arm, D2 on the Manα1-3Manα1-6 arm, D3 on the Manα1-3 arm of M6 and on the Manα1-2Manα1-3 arm of M7 and M8; A*x*, number of antenna (GlcNAc) on trimannosyl core; A2, biantennary with both GlcNAcs as *β*1-2 linked; A3, triantennary with a GlcNAc linked *β*1-2 to both mannose and the third GlcNAc linked *β*1-4 to the α1-3 linked mannose; A4, GlcNAcs linked as A3 with additional GlcNAc *β*1-6 linked to α1-6 mannose; B, bisecting GlcNAc linked*β*1-4 to *β*1-3 mannose; G*x*, number (*x*) of β1-4 linked galactose on antenna; [3]G1 and [6]G1 indicates that the galactose is on the antenna of the α1-3 or α1-6 mannose; F(*x*), number (*x*) of fucose linked α1-3 to antenna GlcNAc; Lac(*x*), number (*x*) of lactosamine (Gal*β*1-4GlcNAc) extensions.
