# Supplementary material for: Genomics Meets Glycomics—The First GWAS Study of Human N-Glycome Identifies HNF1α as a Master Regulator of Plasma Protein Fucosylation
Source: PLoS Genet. 2010 Dec 23;6(12):e1001256. doi: 10.1371/journal.pgen.1001256 (PMC3009678; doi:10.1371/journal.pgen.1001256)
Supplement: Table S2 — Desialylated glycan traits (DG1-13, FUC-A, FUC-C) and their associations with FUT6, FUT8 and HNF1α SNPs showing their effect sizes (Beta) in standard deviation units (with standard errors) and p-values. The fucosylation status of each trait is also shown. (0.06 MB DOC) [file pgen.1001256.s005.doc]

| **Glycan Trait** | | **Fucosylation Status*** | | | **FUT8** | | | | | | | | | | | | | **FUT6** | | | | | | **HNF1Α** | | | | | | | | |
| --- | --- | --- | --- | --- | --- | --- | --- | --- | --- | --- | --- | --- | --- | --- | --- | --- | --- | --- | --- | --- | --- | --- | --- | --- | --- | --- | --- | --- | --- | --- | --- | --- |
|  |  |  |  |  | **rs7159888** | | | | | | **rs10483776** | | | | | | | **rs3760776** | | | | | | **rs7953249** | | | | **rs735396** | | | | |
|  |  |  |  |  | **Beta** | | | ***P*** | | | **Beta** | | | | ***P*** | | | **Beta** | | | ***P*** | | | **Beta** | | | ***P*** | **Beta** | | | | ***P*** |
| **DG1** | | | NF | | | -0.2645 | | | 1.50E-18 | | | 0.2186 | | 8.62E-09 | | | -0.0711 | | | 1.33E-01 | | | -0.0061 | | | 8.38E-01 | | | 0.0385 | | 2.09E-01 | |
| **DG2** | | | CF, NF | | | 0.0072 | | | 8.13E-01 | | | -0.0033 | | 9.31E-01 | | | 0.0353 | | | 4.67E-01 | | | 0.0293 | | | 3.31E-01 | | | 0.0534 | | 8.47E-02 | |
| **DG3** | | | NF, CF | | | -0.0947 | | | 1.98E-03 | | | 0.0381 | | 3.24E-01 | | | -0.0424 | | | 3.81E-01 | | | -0.0178 | | | 5.55E-01 | | | -0.0297 | | 3.38E-01 | |
| **DG4** | | | CF, NF | | | 0.0205 | | | 5.04E-01 | | | -0.0167 | | 6.65E-01 | | | 0.0229 | | | 6.38E-01 | | | 0.0212 | | | 4.84E-01 | | | 0.0539 | | 8.35E-02 | |
| **DG5** | | | NF | | | -0.0310 | | | 3.09E-01 | | | 0.1052 | | 5.99E-03 | | | -0.0293 | | | 5.42E-01 | | | 0.0089 | | | 7.68E-01 | | | -0.0181 | | 5.56E-01 | |
| **DG6** | | | CF | | | 0.0646 | | | 3.51E-02 | | | -0.2242 | | 6.19E-09 | | | -0.0033 | | | 9.46E-01 | | | -0.0337 | | | 2.66E-01 | | | -0.0239 | | 4.43E-01 | |
| **DG7** | | | AF | | | -0.0357 | | | 2.47E-01 | | | -0.0078 | | 8.41E-01 | | | 0.3390 | | | 3.21E-12 | | | -0.1694 | | | 2.72E-08 | | | -0.1747 | | 2.51E-08 | |
| **DG8** | | | NF, CF, AF | | | 0.0121 | | | 6.93E-01 | | | 0.0257 | | 5.06E-01 | | | -0.2144 | | | 1.05E-05 | | | 0.1251 | | | 3.79E-05 | | | 0.1422 | | 5.29E-06 | |
| **DG9** | | | AF, CF | | | -0.0205 | | | 5.06E-01 | | | 0.0123 | | 7.52E-01 | | | 0.4104 | | | 3.51E-17 | | | -0.1662 | | | 5.06E-08 | | | -0.1636 | | 1.83E-07 | |
| **DG10** | | | CF+AF | | | 0.0698 | | | 2.35E-02 | | | -0.1689 | | 1.36E-05 | | | 0.1685 | | | 5.57E-04 | | | -0.0845 | | | 5.53E-03 | | | -0.0656 | | 3.62E-02 | |
| **DG11** | | | NF, CF, AF | | | 0.0036 | | | 9.06E-01 | | | 0.0825 | | 3.24E-02 | | | -0.1480 | | | 2.29E-03 | | | 0.1560 | | | 2.39E-07 | | | 0.1716 | | 3.25E-08 | |
| **DG12** | | | AF | | | -0.0106 | | | 7.30E-01 | | | 0.0331 | | 3.92E-01 | | | 0.2974 | | | 9.44E-10 | | | -0.0896 | | | 3.16E-03 | | | -0.0950 | | 2.32E-03 | |
| **DG13** | | | AF, CF+AF, NF | | | -0.0358 | | | 2.28E-01 | | | 0.0371 | | 3.24E-01 | | | 0.0851 | | | 6.94E-02 | | | -0.0170 | | | 5.64E-01 | | | 0.0010 | | 9.72E-01 | |
| **FUC-A** | | | AF | | | -0.0218 | | | 4.80E-01 | | | -0.0396 | | 3.08E-01 | | | 0.3446 | | | 1.41E-12 | | | -0.1626 | | | 9.12E-08 | | | -0.1643 | | 1.51E-07 | |
| **FUC-C** | | | CF | | | 0.0633 | | | 3.84E-02 | | | -0.2038 | | 1.13E-07 | | | 0.0207 | | | 6.69E-01 | | | -0.0261 | | | 3.86E-01 | | | -0.0089 | | 7.74E-01 | |
|  |  | | |  | | |  | | |  | | |  | | |  | | |  | | |  | | |  | |  | | |  | |  |
|  | * in order of prevalence, NF=non-fucoyslated, CF=core fucosylated, AF= antennary fucosylated, CF+AF = antennary and core fucosylated | | | | | | | | | | | | | | | | | | | | | | | | | | | | | | | |
