# Supplementary material for: Genomics Meets Glycomics—The First GWAS Study of Human N-Glycome Identifies HNF1α as a Master Regulator of Plasma Protein Fucosylation
Source: PLoS Genet. 2010 Dec 23;6(12):e1001256. doi: 10.1371/journal.pgen.1001256 (PMC3009678; doi:10.1371/journal.pgen.1001256)
Supplement: Text S1 — Supplemental Materials and Methods. (0.05 MB DOC) [file pgen.1001256.s006.doc]

**Supplemental Materials and Methods**

# ChIP and reChIP primers

Forward P1 TCTGAAGGTCTGGCCAATCTCACT

Reverse P1 ACACATAGGTGTCTCTCTGAGCCT

Forward P2 GTGTCCTGCTGAGTACACACAACT

Reverse P2 TCAGCCTCCCAAAGTACTGGGATT

Forward P3 GTGGCTCATGCTTGTAATCCCAGT

Reverse P3 CTCTGTCTCCCAGGTTCAAGCAAT

Forward P4 TACTCGGGAGGCTGAGACAAGAGAAT

Reverse P4 GAAGGGCTCATTGGCCACCAATTT

## FUT6/FUT3 promoter/ 3’UTR

Forward P5 TCCCAAGGCTACAACCCTTCTCCTTA

Reverse P5 GATGTTCCTTCATGGCTGGGTGAT

Forward P6: AGCTACAGCTGGAGACACTCAAGG

Reverse P6: GGCCCAAAGGGTGGAGGAAT

Forward P7: TCACACCTGTAGTCCCAGCACTTT

Reverse P7: TGGGTTCAAGTGATTCTCGTGCCT

## FUT3/FUT5 promoter/ 3’UTR

Forward P8: GCATGGGCTTCTAAAGCC

Reverse P8: GCTGGGACAAAGGGTGC

Forward P9: CCCTGGCAGCTCACGG

Reverse P9: CGCAGCTCCTCTTCCAGAT

Forward P10: CAGACCCCCCAGCTCCTCC

Reverse P10: CCGTGGGGCCTTCTCCCT

Forward P11: GGCCTTGGGTGAGTTCCTC

Reverse P11: GTGTGTGGCCTTGTGGATG

## FUT5 promoter

Forward P12: AGCCTCCCAAAGTGCTGGGATTA

Reverse P12: GGAAACTTACCTGGCTCCTTGGACTT

Forward P13: TCTGTGTGCCTCAAAGCCACT

Reverse P13: ACCTGAAGGTGAAATGTGGGCTCT

Forward P14: ACCTTCAGGTATCACGACTTCCCT

Reverse P14: AGAGAACAGACTCAGGTCATCCCT

Forward P15: ACACTTTGAACCTCACGCCTGG

Reverse P15: GGACCACGTGCATTGATTTCTTCAAGG

## GDMS promoter

Forward P16: AGTGAGTTCACTGTTGGCTGG

Reverse P16: ATGGAGGCTTCATTATGTCGGC

Forward P17: TAAGTTGATCACCAACGGCCAACG

Reverse P17: GAGGAGATGTACAGGGCAAGGAAT

Forward P18: GTTCCGTGAGCCACTCTTTCAGAT

Reverse P18: TCAACTCTGACACTGTCTCCTTGG

Forward P19: GGAAACATTAATAAACTCGCCCAAGGC

Reverse P19: GAGCAGGAACGCATCTAATTGACAAAGC

Forward P20: TCTGGCAGGTGGAAGTGAAACAGA

Reverse P20: TACCACCATAGCTGAAGGCCAGAA

## L-Fucokinase promoter

Forward P21: CTTCACTCTGCCACTTGCTAGCTT

Reverse P21: GGGTGTGGTGGCTCATGTCTATAA

Forward P22: GGGCTCAGGAGTCAGATTGGTTT

Reverse P22: ACTTGGAGACCTCCCTTCCCA

Forward P23: AACTTCTGGGCCATTGGACTCTCT

Reverse P23: AAATTAGCCGGGTGTAGTGATGCG

## L-Fucokinase 3’UTR

Forward P24:GGAAGGTCCCAAGCTTAGTATCCCA

Reverse P24: GGTTGAGTGTAAGTCTACAACTCTAAGCCA

Forward P25: TTGCAGGTGGTCCTCGTAAAGTCA

Reverse P25: CCACACACAGTGCAGCACACA

## FUT11 promoter

Forward P26: GGACCTCTCATTTCTCTTCATTGG

Reverse P26: GATAGTCGTGACCGGACGA

Forward P27: CTCCCCACCTGGGATGCCC

Reverse P27: CTGCTTCTCCAGGCCCGGG

**FUT11 enhancer (element 595, chr10:75,847,771-75,849,006 )**

Forward P28: GATGCCTCTGCTCTCCAG

Reverse P28: GACAGCTGCTATAAGTATG

Forward P29: TGTCGATAAACTTCCCAGCTCAGT

Reverse P29: CCACCAAACATTTGGCTCCTGTTC

Forward P30: GAGCTTGCCCTGTAATTTGATCCC

Reverse P30: AGTTCACAATCAGTAGAGTCCATGC

## FUT10 promoter

Forward P31: AGAGCAAGCTTCTGCCATGTGA

Reverse P31: AGTGTCTAGTCCGTTTGGGCTACT

Forward P32: ATCATCCAGACTGGGAGCAGTG

Reverse P32: ACCTCCTTAGGTTTATTACCTCCTAGAC

Forward P33: GCAAGGCAAATGAGGTACTTAGGG

Reverse P33: TGTGGACACCATTATTATCAACCTGT

## HNF4αlpha promoter

Forward P34: TGGACTCTCACCTCTCCAGCC

Reverse P34: TAACCAGTCACTTAGGGAACCCGC

Forward P35: CTCTTTAACGTATCCACCCACCTTGG

Reverse P35: CGCGTTCACGCTGACCAT

Forward P36: GGCTTGGCCATGGTCAGCGTGAA

Reverse P36: GGGCTTCCTGCCCTTCCAGA

**HNF4αlpha 3’UTR**

Forward P37: ACATCCAGGAGGAATAAGCTCCCA

Reverse P37: ACAATCTCTAGGTTAGGAGGGAGTGG

## Real Time primers

| **FUT3F** | ATCCCCTAGGGCTCCCAGTG |
| --- | --- |
| **FUT3R** | TGTCTGCCTGTGGGTACACCT |
| **FUT6F** | GCTTCCCAGACAGCACAGG |
| **FUT6R** | CCTCTCGGTGGTGCACGA |
| **FUT7F** | GAGAAATTCTGGCGCAACGCA |
| **FUT7R** | GGGTAGGTGTGGGTAGCGGTCA |
| **FUT9F** | TGCATTATCCTGGGCTGTTTCA |
| **FUT9R** | GGAAGGGTGGCCTAGCTTGC |
| **HNF4F** | CACCTGATGCAGGAACATATGG |
| **HNF4R** | CTGTCCGTTGCTGAGGTGAGT |
| **HNF1F** | GCAGCCTGGTGCTGTACCA |
| **HNF1R** | GGGAGGAAGAGGCCATCTG |
| **Fut8F** | GTCCATGGTGATCCTGCAGTGTGG |
| **Fut8R** | CACCAATGATATCTCCAGGTTCC |
| **Fut10F** | TCTGTTGGTTTCCAGGGATG |
| **Fut10R** | TCAGGGTATGGAGAAGAAGGTAGA |
| **Fut11F** | TCAGAAGCTGGCAGAGTTTATTG |
| **Futt11R** | CGAAGCCGTTGAGGTAGTTAG |
| **Fut5F** | CGCTGGATCTGGTTCAGC |
| **Fut5R** | CAGCCGTAGGGCGTGAAG |
| **GMDSF** | AGGAAGGTGGCGCTCATCACGGGC |
| **GMDSR** | TAAGGCCACAAGTCTTAATTGCATCC |
| **L-FuKF** | AAGTTGGTAGCAGTGACCCAGTGA |
| **L-FuKR** | AAGAATGCCAGTCCTGAGAACCCA |

## Double stranded RNA for RNA interference

**HNF1αlpha (NM_000545)**

TCAAAGAGCTGGAGAACCT

TTAGTAAAGTCAAGGAGAA

GGCAGATGTAGGAGGGACT

**HNF4αlpha (NM_001030004)**

TGGACAAAGACAAGAGGAA

CGAAGAAGATTGCCAGCAT

TCATCAAGCTCTTCGGCAT
